# Celiac Artery Compression Syndrome

**DOI:** 10.1155/2013/934052

**Published:** 2013-04-04

**Authors:** Mohammed Muqeetadnan, Syed Amer, Ambreen Rahman, Salman Nusrat, Syed Hassan

**Affiliations:** ^1^Department of Medicine, University of Oklahoma Health Sciences Center, Oklahoma City, OK 73104, USA; ^2^Department of Internal Medicine, Brookdale University Hospital and Medical Center, Brooklyn, NY 11212, USA; ^3^Department of Internal Medicine, Henry Ford Hospital, Detroit, MI 48202, USA

## Abstract

Celiac artery compression syndrome is a rare disorder characterized by episodic abdominal pain and weight loss. It is the result of external compression of celiac artery by the median arcuate ligament. We present a case of celiac artery compression syndrome in a 57-year-old male with severe postprandial abdominal pain and 30-pound weight loss. The patient eventually responded well to surgical division of the median arcuate ligament by laparoscopy.

## 1. Introduction

Celiac artery compression syndrome also known as median arcuate ligament syndrome is a rare cause of abdominal pain and weight loss. It is caused by compression of the celiac artery by the median arcuate ligament. We report a case of a 57-year-old male who presented to us with this rare diagnostic challenge. Various imaging techniques such as duplex ultrasound, magnetic resonance angiography, computerized tomography angiogram, and visceral angiography can be used to diagnose this condition. Surgical decompression of the celiac artery by division of the median arcuate ligament has been shown to be very effective.

## 2. Case Report

**Figure 1:**
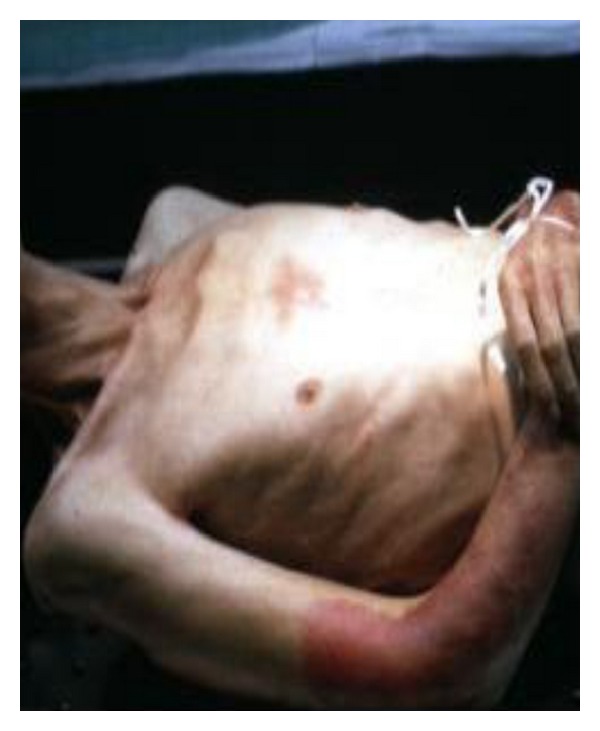
Cachectic patient (as a result of his 30-pound weight loss).

A 57-year-old male with past history significant for hepatitis C and chronic abdominal pain with 50 admissions in various hospitals over the last 2 years presented with worsening epigastric and left upper quadrant pain as well as a 30-pound weight loss over one year (Figure 1). The pain was sharp, constant, 10/10 in severity, and radiating to the back and chest. It was aggravated by deep inspiration and was associated with nausea and diarrhea. He had about 3-4 loose nonbloody bowel movement per day. He also reported decreased appetite. There was no reported history of fever, chills, cough, shortness of breath, or chest pain.

**Figure 2:**
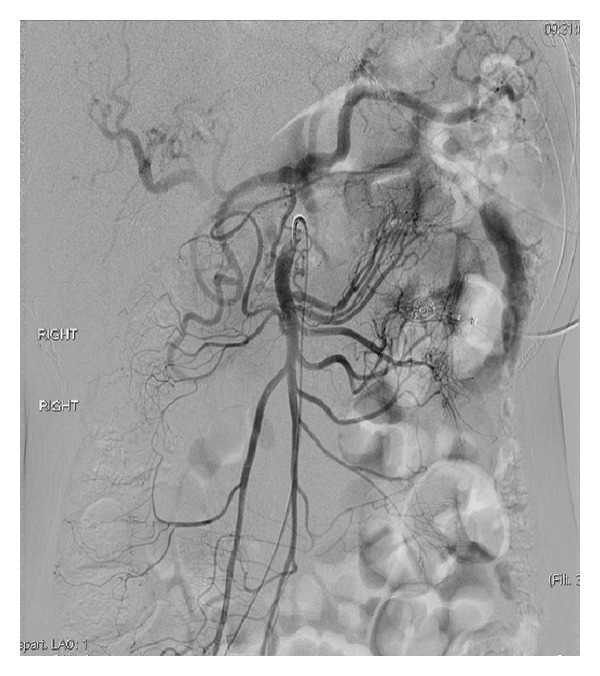
CT angiogram showing increased retrograde flow through the collaterals from superior mesenteric artery to celiac artery.

**Figure 3:**
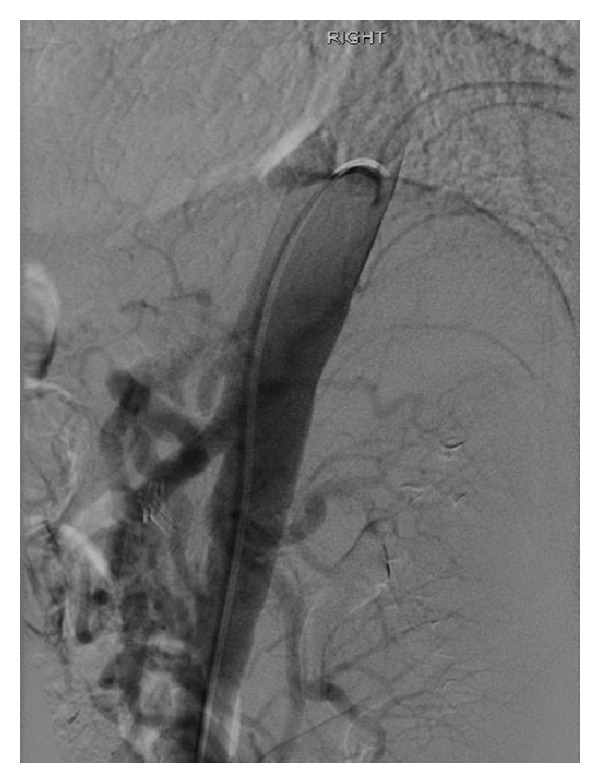
External compression of celiac artery likely by median arcuate ligament.

A detailed exam was notable for cachexia and normal vital signs. S1 and S2 were audible with no murmurs or gallop. Lungs were clear to auscultation. Abdomen was flat, nontender with normal bowel sounds. He had 5/5 strength bilaterally with no sensation abnormalities. He was extensively evaluated in the past; he recently had a normal colonoscopy and esophagogastroduodenoscopy. Endoscopic ultrasound demonstrated a normal common bile duct with no filling defects. Pancreas showed no atrophy, calcification, or pseudocyst. Labs on admission showed normal electrolytes hemoglobin 10 g/dl, platelets 120 × 10^9^/L, and white blood cells 6.8 × 10^9^/L. Liver function tests were elevated (alanine aminotransferase 216 U/L, aspartate aminotransferase 414 U/L, and alkaline phosphatase 191 U/L) likely secondary to Darvocet use, which returned to baseline during his hospital course. Antismooth muscle antibody (ASMA), antinuclear antibodies (ANA) and intrinsic factor blocking antibody were within normal limits. Computed axial tomography (CAT) scan of the abdomen was normal and showed no signs of cirrhosis. Stool hemoccult was positive. Capsule endoscopy was done which showed delayed gastric emptying. Visceral angiogram was done which showed no signs of vasculitis but near complete stenosis of celiac axis secondary to median arcuate ligament compression and retrograde filling of celiac artery (Figures 2 and 3). He was treated with surgical division of median arcuate ligament laparoscopically. Postoperatively, his pain resolved and he started gaining weight too.

## 3. Discussion

Celiac artery compression syndrome is an extremely rare condition. It is also referred to as median arcuate ligament syndrome or Danbury syndrome. Clinically it is characterized by the triad of postprandial abdominal pain, weight loss, and sometimes an abdominal bruit [1]. It occurs as a result of focal stenosis of the celiac artery due to compression by the median arcuate ligament—the bow shaped fibrous arch that bridges the two medial borders of the diaphragmatic crura on either side of the aortic hiatus. This stenosis may arise as a consequence of an abnormal inferior insertion of the diaphragm or an abnormally superior origin of the celiac artery. Reports of celiac artery compression syndrome occurring in several members of the same family by Okten et al. [2] and in monozygotic twins by Bech et al. [3] suggest that congenital factors may play a possible role in its etiology.

Diagnosis remains a challenge. It is a diagnosis of exclusion. Like our patient, most patients with this syndrome have had an extensive workup for other causes of abdominal pain. These patients might undergo unnecessary cholecystectomies and appendectomies in an attempt to relive pain. Diagnostic modalities that are helpful in making a diagnosis include Doppler ultrasound (US), magnetic resonance angiogram (MRA), and computerized tomography angiogram (CTA). However, CT angiogram remains the gold standard. Findings include retrograde filing of celiac artery via gastroduodenal branch of the superior mesenteric artery and poststenotic dilatation.

Various treatment modalities have been tried to decompress the celiac artery. Surgical division of the fibers of the median arcuate ligament either by conventional open surgery (either the retroperitoneal approach via left subcostal incision or the transabdominal approach via a median incision) or by laparoscopic approach is usually adequate in most patients [4]. Recently, the laparoscopic approach has been preferred; as being less invasive it avoids the morbidity of an upper-midline laparotomy and thereby results in shorter hospital stay. The other modalities of treatment [5] that have been tried include celiac artery bypass surgery, transluminal dilation, and stent placement.

## 4. Conclusion

Celiac artery compression syndrome is a rare condition that may present a real diagnostic challenge for the clinician. We presented an unusual case of a 57-year-old male, with the median arcuate ligament compressing on the superior mesenteric artery, thereby causing postprandial abdominal pain and weight loss. Our patient was successfully treated by laparoscopic division of the median arcuate ligament. At least three-fourths of the patients have a good long-term prognosis following the surgical division of the median arcuate ligament [6].
